# Association between chronological depressive changes and physical symptoms in postoperative pancreatic cancer patients

**DOI:** 10.1186/s13030-018-0132-1

**Published:** 2018-09-28

**Authors:** Naoko Sato, Yoshimi Hasegawa, Asami Saito, Fuyuhiko Motoi, Kyohei Ariake, Yu Katayose, Kei Nakagawa, Kei Kawaguchi, Shin Fukudo, Michiaki Unno, Fumiko Sato

**Affiliations:** 1 Department of Oncology Nursing, Tohoku University Graduate School of Medicine, Sendai, Japan; 2 Department of Nursing, Tohoku University School of Health Sciences, Sendai, Japan; 3 Department of Surgery, Tohoku University Graduate School of Medicine, Sendai, Japan; 4 Department of Behavioral Medicine, Tohoku University Graduate School of Medicine, Sendai, Japan; 5 Department of Oncology Nursing, Tohoku University, 2-1 Seiryo-machi, Aoba-ku, Sendai, 980-8575 Japan

**Keywords:** Pancreatic cancer, Depression, Physical symptoms

## Abstract

**Background:**

Pancreatic cancer (PC) has poorer prognosis and higher surgical invasiveness than many other cancers, with associated psychiatric symptoms including depression and anxiety. Perioperative depression has not been investigated in PC patients regarding surgical stress and relevant interventions.

**Methods:**

We evaluated chronological depressive changes and subjective physical symptoms in surgically treated PC patients preoperatively and at 3 and 6 months postoperatively.

Enrolled patients undergoing pancreatic tumor surgery completed questionnaires based on the Self-Rating Depression Scale (SDS) and Functional Assessment of Cancer Therapy for Patients with Hepatobiliary Cancer (FACT-Hep) preoperatively, and at 3 and 6 months postoperatively. Responses were analyzed with JMP® Pro using one-way and two-way ANOVA, Spearman’s rank correlation coefficient, and multiple regression analysis.

**Results:**

Malignancy was diagnosed in 73 of 101 patients postoperatively; SDS score was significantly higher in these patients than in those with benign tumors at all timepoints: malignant/benign, 41.8/37.9 preoperatively (*p* = 0.004); 43.5/37.8 3 months postoperatively (*p* = 0.006); and 42.9/37.7 6 months postoperatively (*p* = 0.020). SDS scores were significantly higher in patients < 65 years old with malignancy at 3 months than at 6 months postoperatively (44.6/42.5, *p* = 0.046) and in patients with malignancy who underwent pancreaticoduodenectomy at 3 months postoperatively than preoperatively (43.4/41.1; *p* = 0.028). SDS scores moderately correlated with 8 physical symptom-related FACT-Hep items 3 months postoperatively (*p* < 0.05), showing low-to-moderate correlation with 16 physical symptom-related FACT-Hep items at 6 months postoperatively (*p* < 0.05). Multiple regression analysis of FACT-Hep symptoms significantly correlated with SDS scores revealed the following significant variables: “lack of energy” (*p* < 0.000) and “pain” (*p* = 0.018) preoperatively (R^2^ = 0.43); “able to perform usual activities” (*p* = 0.031) and “lack of energy” (*p* < 0.000) at 3 months postoperatively (R^2^ = 0.51); and “stomach swelling or cramps” (*p* = 0.034) and “bowel control” (*p* = 0.049) at 6 months postoperatively (R^2^ = 0.52).

**Conclusions:**

PC patients experience persistently high levels of depression preoperatively through 6 months postoperatively, with associated subjective symptoms including pain and gastrointestinal symptoms.

**Trial registration:**

UMIN Clinical Trials Registry 000009592, Registered 20 December 2012.

## Background

Pancreatic cancer (PC) has a poorer prognosis than other cancers [1]. Although the 5-year survival rate in patients with localized cancer at the time of detection has increased to 30% following advances in medical science, more than 50% of PC patients have distant metastases when the cancer is diagnosed, and the overall survival rate for PC remains low, at 8% [1]. Surgical resection is associated with a higher survival rate for PC [2] and is currently the sole treatment by which complete cure might be expected [3]. However, such treatment entails a high level of surgery-related stress and is associated with high rates of postoperative mortality and complications [4–7].

Pancreatic tumor (PT) resection causes high surgical stress regardless of whether the tumor is benign or malignant, and deterioration of quality of life (QOL) is a concern even when a benign tumor is diagnosed based on postoperative histology [5]. In light of concern over future malignant conversion, health professionals should consider a common approach to the physical and mental health of pancreatic resection patients facing surgical stress in the perioperative period, with no separate distinction made for those that receive a diagnosis of malignancy.

We reported on the extent of surgical stress in pancreatic resection and symptoms necessitating intervention in a previous study [8]. Among postoperative symptoms, loss of appetite, general fatigue, and pain correlated with deterioration in QOL [8]. In previous studies, PC patients with depression reportedly showed associations between significantly aggravated symptoms of fatigue, pain, and anorexia with QOL deterioration [9], and depression in PC patients was considered to be part of the symptomatology with an impact on QOL [10]. There are numerous previous reports on the psychological state of patients for a range of cancers [11–13]. The Reports from studies of PC patients undergoing treatment are significantly important. Though some assessment of the QOL of PC patients has been reported [3, 9, 14–20], no reports show an association between chronological depressive changes and physical symptoms in PC patients postoperatively.

In this study, we aimed to elucidate associations between depressive changes and self-reported, subjective physical symptoms with chronological assessments of depression in PT resection patients pre-operatively, and at 3 and 6 months postoperatively, by a prospective investigation.

## Methods

### Participants

This study is a part of an analysis of an observational study within a prospective cohort study on the correlation between corticotropin-releasing hormone expression and PT prognosis, with a QOL survey. Therefore, the hypothesis of this study and the hypothesis of the main study are completely different. We prospectively recruited patients diagnosed with PT and hospitalized for surgical treatment between January 2103 and December 2015 to obtain assessments of depressive and physical symptoms preoperatively, and postoperatively at months 3 and 6. The analysis set consisted of 101 tumor-resection patients who underwent pancreaticoduodenectomy (PD), distal pancreatectomy (DP), or total pancreatectomy (TP), excluding patients who underwent exploratory bypass laparotomy and non-resected cases.

### Materials

The Self-Rating Depression Scale (SDS) [21, 22] was used to assess depression. The SDS assessment form has 20 items, each of which is rated with a score of 1 to 4 (rarely; sometimes; frequently; always) with higher scores indicating higher levels of depression. According to Zung, the cutoff point for depression is 40. The SDS is used to measure depression in cancer patients [23–26].

Physical symptoms were assessed using the Functional Assessment of Cancer Therapy for Patients with Hepatobiliary Cancer (FACT-Hep) [27, 28], which provides scales for rating responses with regard to treatment and disease progression for hepato-biliary-pancreatic cancers. The FACT-Hep items used were the 18 items from the Hepatobiliary Cancer Subscale (HCS) and three of seven items from the Physical Well-Being (PWB) subscale (hereinafter, “physical symptoms”). Generally, higher HCS and PWB scores indicate better physical condition; however, in this study, we inverted the scores for the relevant HCS (Questions 1, 2, 5, 7, 8, 9, 10, and 12 to 18) and PWB (Questions 19 to 21) items to align them with SDS score, which indicates awareness of symptoms. We presented the details in raw form for consistent display of higher scores indicating worse symptoms in this study.

### Data analysis

In this study, the primary endpoint was the chronological depressive score of perioperative PC patients, and secondary endpoints were physical symptoms related to chronological depression.

Chronological changes in SDS scores were compared preoperatively and postoperatively at 3 and 6 months with one-way or two-way ANOVA tests. Patient demographics and physical symptom scores were independent variables and SDS score was a dependent variable. SDS scores of patients with malignant tumors based on histology were further analyzed by categories. When significant interactions were detected, post hoc multiple comparisons were made using the Bonferroni method. Associations between physical symptom scores and SDS score were evaluated with Spearman’s rank correlation coefficient. Correlation coefficients (r_s_) were interpreted as follows: a magnitude of r between 0.7 and 1.0 indicated “high correlation”; a magnitude of r between 0.4 and 0.7 indicated “moderate correlation”; a magnitude of r between 0.2 and 0.4 indicated “low correlation”, and a magnitude of r below 0.2 indicated “substantially no correlation”. Multiple regression analysis was applied where coefficients were significant in univariate analysis. Symptoms were subjected to entity-relationship modeling preoperatively and postoperatively at 3 and 6 months, with regard to patient symptoms as independent variables and SDS score for the relevant time point as a dependent variable. The distribution of the SDS variable was checked for normality using the Kolmogorov-Smirnov test. All statistical analyses were performed using JMP Pro 12 software (Ver. 12, SAS Institute, Milan, Italy); *p* values less than 0.05 were considered significant.

## Results

### Patient demographics and chronological changes in SDS score

Subjects were a group of patients being treated for PT. SDS scores were the highest at 3 months after surgery with surgical invasion in many patients.

The analysis set of 101 patients had a mean age of 63.3 ± 10.7 (28 to 82) years [53 men (52.5%) and 48 women (47.5%)]. Postoperative histology revealed 73 patients (72.3%) with malignant tumors and 28 patients (27.7%) with benign tumors. SDS scores for the 101-patient analysis set were 40.8 preoperatively, 41.9 at postoperative month 3, and 41.5 at postoperative month 6. Patients undergoing PD had a significantly higher SDS score at postoperative month 3 than pre-operation (43.2 vs. 40.9. *p* = 0.016). Patients with malignant tumors had significantly higher SDS scores at each time point than patients with benign tumors (pre-operation: 41.9 vs. 37.9, *p* = 0.004; postoperative month 3: 43.5 vs. 37.8, *p* = 0.006; postoperative month 6: 42.9 vs. 37.7, *p* = 0.020). SDS scores of patients with malignant tumors were further analyzed by category, with results as follows. Malignant PT patients under 65 years old showed a significantly higher SDS score at postoperative month 3 than postoperative month 6 (44.6 vs. 42.5, *p* = 0.046). Malignant PT patients undergoing PD showed a significantly higher SDS score at postoperative month 3 than pre-operation (43.4 vs. 41.1, *p* = 0.028) (Table 1).

**Table 1:** Patient characteristics and SDS scores n=101

		n (%)	Preop	3 month postop	6 month postop
Age, years	under 65	47 (46.5)	41.32	42.87	41.94
	65 or over	54 (53.5)	40.26	41.07	41.06
Sex	male	53 (52.5)	40.28	42.00	42.40
	female	48 (47.5)	41.27	41.81	40.44
Operation	PD	48 (47.5)	40.85	43.21 †	42.23
	DP	42 (41.6)	40.83	39.52	39.93
	TP	11 (10.9)	40.00	45.36	44.00
Clavian_Dindo	0	24 (23.8)	38.92	39.58	40.17
	I	18 (17.8)	42.83	41.83	41.67
	II	26 (25.7)	40.15	41.12	39.23
	III a	28 (27.7)	42.71	45.25	46.07
	III b	3 (3.0)	35.00	39.67	32.00
	IV a	2 (2.0)	33.00	37.50	34.00
Histology	benign	28 (27.7)	37.89	37.75	37.71
	malignant	73 (72.3)	41.85 **	43.51 **	42.92 *
	under 65	32 (43.8)	42.56	44.59	42.47 ‡
	65 or over	41 (56.2)	41.29	42.66	43.24
	male	42 (57.5)	40.83	43.19	42.93
	female	31 (42.5)	43.23	43.94	42.87
	PD	41 (56.2)	41.15	43.44†	42.98
	DP	23 (31.5)	43.52	42.39	41.83
	TP	9 (12.3)	40.78	46.67	45.33
R status	R0	66 (90.4)	41.88	43.27	42.86
	R1	7 (9.6)	41.57	45.71	43.29

Compared with benign * *p*<0.05 **p<0.01

Compared with preoperatively † *p*<0.05

Compared with 3 months postoperatively‡ *p*<0.05

*PD* subtotal stomach-preserving pancreatoduodenectomy, *DP* distal pancreatectomy, *TP* total pancreatectomy

### Chronological changes in subjective physical symptoms

Subjective physical symptoms at postoperative month 3 were worse compared with preoperatively, but many of the symptoms showed improvement at postoperative month 6. Detailed results for subjective physical symptoms are described below.

**Fig. 1:**
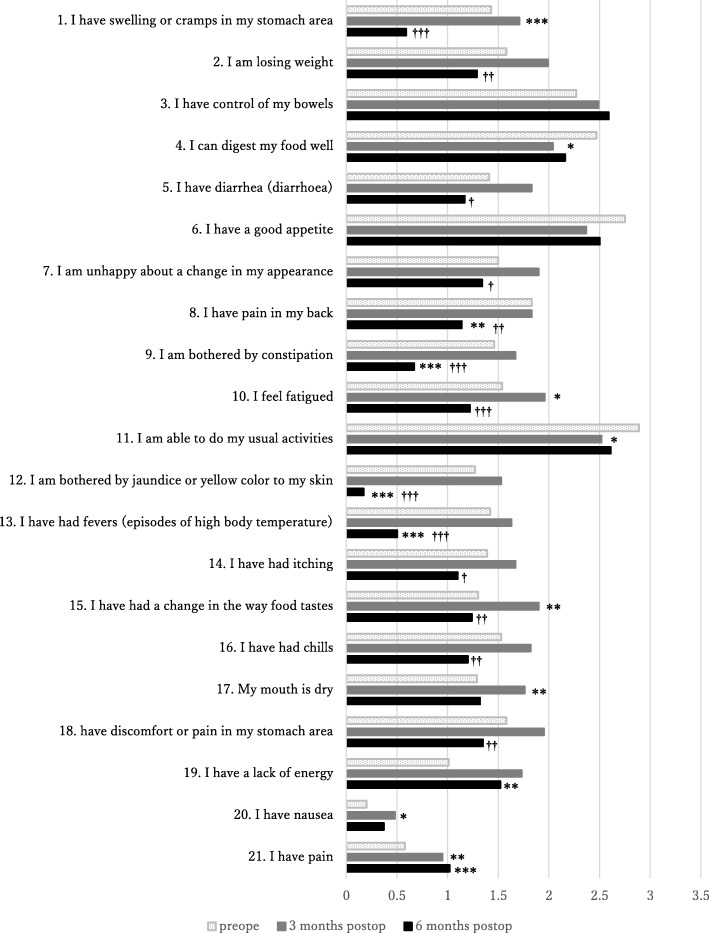
Chronological changes in subjective physical symptoms scores preoperation, post 3 and 6 months operation. Scores were reversed for items 1, 2, 5, 7, 8, 9, 10, 12, 13, 14, 15, 16, 17, 18, 19, 20, and 21. High symptom score was unified to show poor condition. Compared with preoperation * *p* < 0.05, **p < 0.01, ***p < 0.001. Compared with post 3 month operation † p < 0.05, †† p < 0.01, †††p < 0.001. Subjective physical symptoms at postoperative month 3 were worse compared with preoperatively, but many of the symptoms showed improvement at postoperative month 6

Hepato-biliary-pancreatic axis physical symptoms: Abilities related to digestion and performing usual activities (scores for “I can digest my food well” and “I am able to do my usual activities”, respectively) were significantly improved at postoperative month 3 versus preoperatively (*p* < 0.05). Many items showed significant deterioration in physical symptoms at postoperative month 3 versus preoperatively; these were the scores for “I have swelling or cramps in my stomach area” (*p* < 0.001), “I feel fatigued” (*p*<0.05), and “I have had a change in the way food tastes” (*p* < 0.05). Each of these three items then showed significant improvement at postoperative month 6 versus postoperative month 3 (*p* < 0.001, *p* < 0.001, and *p* < 0.01, respectively). Scores for the following items were significantly improved at postoperative month 6 versus postoperative month 3 (after they had tended to show deterioration in physical symptoms at postoperative month 3): “I am losing weight” (*p* < 0.01), “I have diarrhea” (*p* < 0.05), “I am unhappy about a change in my appearance” (*p* < 0.05), “I am bothered by constipation” (*p* < 0.001), “I am bothered by jaundice or yellow color to my skin” (*p* < 0.001), “I have had fevers” (*p* < 0.001), “I have had chills” (*p* < 0.01), and “I have discomfort or pain in my stomach area” (*p* < 0.01) (Fig. 1)

General physical symptoms: Scores for “I have a lack of energy” (*p* < 0.01) and “I have pain” (*p* < 0.01)] showed significant deterioration at postoperative month 3. Both these items showed significant improvement at postoperative month 6 versus postoperative month 3 (*p* < 0.001 for both items) (Fig. 1)

### Associations between physical symptoms and SDS score

Many time-dependent associations were found between physical symptoms and SDS score.

In detail, at pre-operation, the scores for the following six items showed significant correlations with SDS score “I have control of my bowels”, “I can digest my food well”, “I am able to do my usual activities”, “I have a lack of energy”, “I have nausea”, and “I have pain”; these correlations were graded as “low” to “moderate” (*p* < 0.000 – *p* < 0.05). At postoperative month 6, the scores for the following six items showed significant correlation with SDS score: “I can digest my food well”, “I have a good appetite”, “I am able to do my usual activities”, “I have a lack of energy”, “I have nausea”, and “I have pain”; these correlations were graded as “low” to “moderate” (*p* < 0.000 – *p* < 0.05). At postoperative month 6, the following 16 items showed a significant correlation with SDS score: “I have swelling or cramps in my stomach area”, “I am losing weight”, “I have control of my bowels”, “I can digest my food well”, “I have a good appetite”, “I am unhappy about a change in my appearance”, “I feel fatigued”, “I am able to do my usual activities”, “I have had fevers”, “I have had itching”, “I have had a change in the way food tastes”, “I have had chills”, “My mouth is dry,”, “I have discomfort or pain in my stomach area”, “I have a lack of energy”, and “I have pain”; these correlations were graded as “low” to “moderate” (*p* < 0.000 – *p* < 0.05) (Table 2)

**Table 2:** Correlation between symptoms and SDS score

	SDS (r_s_)	3 M SDS (r_s_)	6 M SDS (r_s_)
1. I have swelling or cramps in my stomach area	−.016	−.070	.384***
2. I am losing weight	−.065	−.117	.370***
3. I have control of my bowels	−.291**	−.165	−.329***
4. I can digest my food well	−.255*	−.330***	−.436***
5. I have diarrhea (diarrhoea)	−.070	−.043	.130
6. I have a good appetite	−.170	−.378***	−.354***
7. I am unhappy about a change in my appearance	−.031	.038	.274**
8. I have pain in my back	.041	−.143	.073
9. I am bothered by constipation	.011	−.034	.060
10. I feel fatigued	−.063	.002	.538***
11. I am able to do my usual activities	−.295**	−.400***	−.330***
12. I am bothered by jaundice or yellow color to my skin	−.044	−.099	.104
13. I have had fevers (episodes of high body temperature)	−.011	−.034	.205*
14. I have had itching	−.005	−.031	.220*
15. I have had a change in the way food tastes	−.031	.039	.292**
16. I have had chills	.025	−.102	.287**
17. My mouth is dry	−.056	−.084	.284**
18. have discomfort or pain in my stomach area	.006	.031	.392***
19. I have a lack of energy	.567***	.659***	.498***
20. I have nausea	.209*	.277**	.159
21. I have pain	.449***	.370***	.348***

Spearman’s rank correlation coefficient (r_s_) *p < 0.05 **p < 0.01 ***p < 0.001

Physical symptoms that were significantly correlated with SDS score were subjected to multiple regression analysis, and the results are stated below.

Preoperatively, significant associations were noted for the following two items: “I have a lack of energy” (*p* < 0.000) and “I have pain” (*p* = 0.018) [R^2^ = 0.43; Table 3].

**Table 3:** Model of relationship between symptoms and depression preoperatively

	B	β	*P* value	95% CI [LL, UL]
	41.12		<.000	[36.34, 45.90]
3. I have control of my bowels	−0.90	− 0.14	0.110	[− 2.01, 0.21]
4. I can digest my food well	0.10	0.01	0.876	[1.37, − 1.17]
11. I am able to do my usual activities	−1.11	−0.15	0.076	[0.12, −2.33]
19. I have a lack of energy	3.26	0.42	<.000	[4.58, 1.94]
20. I have nausea	1.31	0.02	0.859	[2.83, −2.36]
21. I have pain	2.20	0.22	0.018	[4.01, 0.39]
R^2^ = 0.43, *p* < .0001				

*CI* confidence interval, *LL* lower limit, *UL* upper limit

At postoperative month 3, significant associations were noted for the following two items: “I am able to do my usual activities” (*p* = 0.031) and “I have a lack of energy” (*p* < 0.000) [R^2^ = 0.51; Table 4].

**Table 4:** Model of relationship between symptoms and depression at 3 months postoperatively

	B	β	*P* value	95% CI [LL, UL]
	37.78		<.000	[31.69, 43.88]
4. I can digest my food well	−0.76	−0.10	0.269	[−2.16, 0.60]
6. I have a good appetite	−0.02	−0.002	0.982	[1.48, 1.44]
11. I am able to do my usual activities	−1.44	−0.17	0.031	[−2.75, −0.14]
19. I have a lack of energy	4.32	0.52	<.000	[2.97, 5.67]
20. I have nausea	0.85	0.07	0.387	[−1.09, 2.78]
21. I have pain	1.55	0.14	0.076	[−0.17, 3.26]
R^2^ = 0.51, *p* < .0001				

*CI* confidence interval, *LL* lower limit, *UL* upper limit

At postoperative month 6, significant associations were noted for the following two items: “I have swelling or cramps in my stomach area” (*p* = 0.034) and “I have control of my bowels” ((*p* = 0.049) [R^2^ = 0.52; Table 5].

**Table 5:** Model of relationship between symptoms and depression at 6 months postoperatively

	B	β	*P* value	95% CI [LL, UL]
	41.41		<.000	[35.54, 47.27]
1. I have swelling or cramps in my stomach area	2.52	0.23	0.034	[0.19, 4.86]
2. I am losing weight	1.22	0.18	0.050	[−0.00, 2.44]
3. I have control of my bowels	−1.46	−0.19	0.049	[−2.91, − 0.01]
4. I can digest my food well	−0.88	− 0.11	0.293	[− 2.52, 0.77]
6. I have a good appetite	−0.82	--0.11	0.263	[−2.25, 0.62]
7. I am unhappy about a change in my appearance	−0.15	−0.02	0.845	[− 1.63, 1.34]
10. I feel fatigued	1.80	0.20	0.089	[−0.28, 3.87]
11. I am able to do my usual activities	−0.10	−0.01	0.890	[−1.60, 1.39]
13. I have had fevers (episodes of high body temperature)	−0.84	− 0.08	0.425	[−2.93, 1.25]
14. I have had itching	0.81	0.010	0.266	[−0.63, 2.24]
15. I have had a change in the way food tastes	−0.51	−0.08	0.467	[−1.91, 0.88]
16. I have had chills	−0.02	− 0.00	0.980	[−1.46, 1.43]
17. My mouth is dry	0.26	0.04	0.730	[−1.24, 1.76]
18. I have discomfort or pain in my stomach area	0.42	0.05	0.641	[−1.38, 2.23]
19. I have a lack of energy	1.34	0.17	0.151	[−0.50, 3.19]
21. I have pain	0.20	0.02	0.844	[−1.82, 2.22]
R^2^ = 0.52, *p* < .0001				

*CI* confidence interval, *LL* lower limit, *UL* upper limit

## Discussion

### Characteristics of depression in PT resection patients

The PT patients in this study were generally considered to be persistently depressed preoperatively through postoperative month 6. In particular, patients who received a postoperative histological diagnosis of malignancy were highly depressed; this finding possibly reflected concerns over the diagnosis, in the same way as has been reported for patients with other cancers [10, 29, 30]. PC has a poor prognosis, and survival rates are poor even when operable [1]. Intraductal papillary mucinous neoplasm is a risk factor for PC even though such tumors are benign [31–33]. Many patients in this study were warned of the likelihood of a malignant tumor prior to undergoing surgery, and the patients who received such a warning might have felt psychological distress concerning the operation and their future and developed tendencies to depression. Even patients who can resume normal living postoperatively could have had fears of relapse and metastasis. In addition to those fears, they may feel a sense of loss and loneliness in their daily lives, and effects could have been attributable to the resultant psychological experience [10, 34]. Levels of depression were high among working-age (under 65 years old) patients with malignant tumors at postoperative month 3, and anxiety about the future has been suggested as likely in this group of cancer patients [35].

### Chronological changes in associations between depression and physical symptoms

The symptoms related to the depression of patients with pancreatic tumor were chronologically distinctive, which may reflect the physical and mental conditions experienced time dependently from surgery. PT patients scheduled for surgery showed depression preoperatively, and this depression was influenced by lack of energy and pain (physical symptom scores for “I have a lack of energy” and “I have pain”, respectively.) Inability to perform usual activities and lack of energy (scores for “I am able to do my usual activities” and “I have a lack of energy”, respectively) were influencing factors for postoperative depression during the period when surgery-related stress was immense. Gastrointestinal symptoms were the main physical symptoms with delayed manifestation, and were suggested to be possible subsequent causative factors for depression. Postoperative depression was high in patients who underwent PD and at postoperative month 3; items related to gastrointestinal symptoms and digestion, and those reflecting surgical stress showed increased scores at postoperative month 3. Accordingly, it was suggested that the extent of surgery-related stress and postoperative physical symptoms possibly affected psychological state. PD involves extensive surgical techniques, prolonged duration of surgery, and a tendency for massive hemorrhage. Likely postoperative complications include intractable diarrhea, pancreatic exocrine insufficiency, and gastrointestinal dysfunction, depending on the extent of the resection [36–39]. Patients with malignant tumors may receive adjuvant chemotherapy when necessary [40, 41], which imposes additional stress to that from surgery. As a result, patients frequently experience toxic effects and manifest gastrointestinal symptoms such as diarrhea and anorexia [41, 42]. These physical symptoms were also suggested to have possibly influenced patients’ psychological state in this study. Most subjective physical symptoms in this area showed dramatic improvement at postoperative month 6. However, we considered that this improvement was due to gradual physical and mental adaption to daily life by the patients, control of blood glucose, and dietary intake amount and content suitable for the patient in question, as well as being an improvement in gastrointestinal symptoms. However, we identified one point that requires attention. The greatest number of associations between subjective physical symptoms and depression was noted at postoperative month 6. We suggest that approaches to monitor and counteract depression are particularly necessary for patients with persistent symptoms 6 months postoperatively and those with severe symptoms.

### Implications

This study demonstrates that surgical invasiveness possibly affects depression in patients with PC. PT patients are reported to often experience fatigue, lower back pain, and digestive system dysfunction [43]. We also identified these physical symptoms in this study and elucidated them as factors that influence depression. Depression in cancer patients generally leads to impairment of QOL, and is linked to more than just physical symptoms, anxiety, and pain; it is associated with reduced adherence to cancer therapy and prolonged hospital stays [10], with the imposition of mental burden on caregivers [44]. Measures for preventing and dealing with depression in cancer patients are extremely important. Depression is reportedly greater in PC patients [9, 45–47] than those with advanced gastrointestinal carcinomas such as stomach and colorectal cancer [48]. Approaches can cover a number of specific points. A symptom management approach that emphasizes gastrointestinal symptoms should be initiated prior to surgery. Care and drug therapies for alleviating these physical symptoms should be provided in the long term as required. Patients should be encouraged to engage in more physical activity [49]. Depression is closely related to QOL and it has been reported that depression interventions including psychoeducational interventions improve QOL [19, 50]. Care that involves supportive-expressive discussion [51], counseling, and psychological support [50, 52] should be provided. Attention to these measures may promote alleviate psychological symptoms and improved physical condition [19]. Based on these reports and the findings in this study, we suggest that proactive symptom management and psychological support is vital for PT patients, especially those with malignant tumors.

### Limitations

First, pancreatic resection involves a high degree of surgery-related stress. In this study, we elucidated the influence of subjective physical symptoms of that stress on depression. Among the patients we evaluated, patients with histologically diagnosed malignant tumors were significantly more depressed than patients with benign tumors. We consider that prognosis-related anxiety may be particularly reflected in depression when the tumor is malignant. PC has a poor prognosis, and the psychological burden should be fully considered in any evaluation. Second, we could not evaluate the influence of diabetes mellitus and insulin supplementation on depression. Future studies with long-term follow up should thus be carried out and to assess long-term effects including QOL. Third, for many patients PC is detected too late for surgery to be effective, and survival is limited even for patients who undergo surgery. There are limits on the parameters that can be investigated in a single center, and a detailed investigation was not possible in this study. Further investigations using multi-center studies are needed for future research on depression in PC patients.

## Conclusions

PC patients experience persistently high levels of depression preoperatively through 6 months postoperatively. Psychological support is needed both pre- and postoperatively, especially for patients with malignant tumors, those undergoing PD, and those under 65 years of age. Several physical symptoms are aggravated due to pancreatic resection between the preoperative stage and 3 months postoperatively, and then are improved at 6 months postoperatively. Pain and gastrointestinal symptoms are the major subjective physical symptoms possibly associated with depression. We consider that depression in PC patients could be reduced by ensuring long-term symptom management commencing prior to surgery and by psychiatric interventions tailored to patient characteristics.

## Abbreviations

DP: Distal pancreatectomy
PD: Subtotal stomach-preserving pancreatoduodenectomy
TP: Total pancreatectomy
